# Heat Shock Protein 70 Improves In Vitro Embryo Yield and Quality from Heat Stressed Bovine Oocytes

**DOI:** 10.3390/ani11061794

**Published:** 2021-06-16

**Authors:** Konstantina Stamperna, Themistoklis Giannoulis, Eleni Dovolou, Maria Kalemkeridou, Ioannis Nanas, Katerina Dadouli, Katerina Moutou, Zissis Mamuris, Georgios S. Amiridis

**Affiliations:** 1 Department of Obstetrics and Reproduction, Veterinary Faculty, University of Thessaly, 43100 Karditsa, Greece; konstantina.stamperna@gmail.com (K.S.); entovolou@uth.gr (E.D.); gnsnanas@gmail.com (I.N.); katerina1dad@gmail.com (K.D.); 2 Department of Animal Sciences, University of Thessaly, 41500 Larissa, Greece; themisgia@gmail.com; 3 Department of Genetics, Comparative and Evolutionary Biology, Faculty of Biochemistry and Biotechnology, University of Thessaly, 41500 Larissa, Greece; mkalemkeridou@uth.gr (M.K.); kmoutou@uth.gr (K.M.); zmamur@uth.gr (Z.M.); 4 Laboratory of Hygiene and Epidemiology, Faculty of Medicine, University of Thessaly, 41500 Larissa, Greece

**Keywords:** HSP70, heat stress, in vitro embryos, gene expression, cattle

## Abstract

**Simple Summary:**

The Holstein cows are among the most thermosensitive farm animals. In this breed, during the heat stress periods, fertility is seriously compromised due to induced alterations of the endocrine status, reduced fertilizing capacity of the oocyte and increased embryo deaths. To combat the deleterious effects of stress, cells synthesize a series of specific molecules that are mainly involved in cellular protection against the heat insult, called heat shock proteins (HSPs). Here, we examined the effects of supplementing HSP70 in in vitro matured bovine oocytes under thermoneutral or heat stress conditions, and we assessed its efficacy on in vitro embryo yield and quality; the latter was determined on the basis of the expression of various genes related to important cellular functions. It was manifested that HSP70 addition into the in vitro maturation medium restores the developmental competence of heat stressed oocytes and improves the quality of the in vitro produced embryos.

**Abstract:**

Heat shock protein 70 (HSP70) is a chaperon that stabilizes unfolded or partially folded proteins, preventing inappropriate inter- and intramolecular interactions. Here, we examined the developmental competence of in vitro matured oocytes exposed to heat stress with or without HSP70. Bovine oocytes were matured for 24 h at 39 °C without (group C39) or with HSP70 (group H39) and at 41 °C for the first 6 h, followed by 16 h at 39 °C with (group H41) or without HSP70 (group C41). After insemination, zygotes were cultured for 9 days at 39 °C. Cleavage and embryo yield were assessed 48 h post insemination and on days 7, 8, 9, respectively. Gene expression was assessed by RT-PCR in oocytes, cumulus cells and blastocysts. In C41, blastocysts formation rate was lower than in C39 and on day 9 it was lower than in H41. In oocytes, HSP70 enhanced the expression of three HSP genes regardless of incubation temperature. HSP70 at 39 °C led to tight coordination of gene expression in oocytes and blastocysts, but not in cumulus cells. Our results imply that HSP70, by preventing apoptosis, supporting signal transduction, and increasing antioxidant protection of the embryo, protects heat stressed maturing bovine oocyte and restores its developmental competence.

## 1. Introduction

Climate change figures at the top of the challenge list, and could have a potentially devastating impact on the global ecosystem and animal welfare. Due to decreased thermoregulatory capacity, dairy cows are particularly vulnerable to heat stress. During summer heat stress, the dry matter intake is reduced and the general physiology of the cow is disturbed, leading to a significant decrease in production and fertility; these seriously compromise the sustainability of the dairy industry and the welfare of the animals [1,2]. The maturing oocyte is particularly sensitive to heat stress in a stage-dependent manner. The sensitivity culminates at the second meiotic arrest during metaphase II [3]. Heat stress induces mitochondrial dysfunction, accumulation of reactive oxygen species and increased apoptosis that inhibits the completion of meiosis I and eventually reduces the developmental competence of the oocyte [4,5,6]. The expression of heat shock proteins (HSPs) is considered the major response mechanism, which the cells operate to maintain their homeostasis against temperature changes [7].

Heat shock protein 70 (HSP70) is a molecular chaperon that protects oocyte against the harmful effects of stress. In mammalian cells, the HSP70 family exists in two isoforms: a constitutively form (HSC70) and a heat-inducible form (HSP70). HSPs were initially associated with the response to heat stress; however, it is now well known that a variety of stressors induce their expression, and therefore they are characterized as “cell stress” proteins [8]. They are normally presented in all cell compartments, such as the cytoplasm, mitochondria, nucleus, endoplasmic reticulum, while during and after heat stress they concentrate mainly in the nucleus [9]. Under normal conditions, HSP70 participates in many important functions such as post translational folding and transportation of cellular proteins through the membranes [10,11]. There is evidence that HSP70 is also involved in fertilization and early embryo development [12]. The addition of antibodies for HSP70 in early embryos cultured in vitro suppresses the blastocyst formation rate both in cattle and in mice [13,14]. Under heat stress, HSP70 plays a vital role by preserving the stability of the cytoskeleton, regulating the cell cycle and the immune response, preventing cell apoptosis and contributing to the thermotolerance of cells [11,15]. Apoptosis is blocked through the interruption of the mechanism of caspase 3 activity [16] as well as by decreasing the phosphorylation of elF-2a, an essential factor for the initiation of protein translation, when reduced protein synthesis during heat stress is needed [15].

Under stress conditions, HSPs are also located in the extracellular space [17], where they regulate functions such as inflammation or acute immune response [18,19]. The secretion of HSPs in the extracellular space can be mediated through a mechanism involving the lysosomes [20] via membrane-bounded particles [21] or by passive leaking from cells undergoing necrotic death, after the disruption of their membranes [17]. Extracellular HSPs are regulators of inflammation [22,23,24] and their regulatory effect is mediated by Toll-Like Receptors (TLRs), particularly TLR2 and TLR4 [18].

In a recent study [25], we have shown that exposure of in vitro maturing oocytes for only 6 h at 41 °C during the early stages of in vitro maturation disorganizes the expression of many genes in the oocytes, the cumulus cells and the blastocyst; in addition, it impairs embryo yield. Here we sought to examine whether the addition of HSP70 in the in vitro maturation medium would prevent the negative effects that a short-term temperature rise causes to the oocyte and to the embryo production rate and quality and how its effect is mediated by key genes.

## 2. Materials and Methods

### 2.1. In Vitro Embryo Production

Unless otherwise stated, all chemicals were purchased from Sigma Chemical Company (Poole, UK). The techniques for in vitro embryo production have been previously described [26,27]. In brief, ovaries from slaughtered mature cows of different breeds (Holstein, Limousine and Holstein crossbreeds) were collected from a local abattoir. The ovaries were transported to the laboratory within 2 h from slaughter, at 37 °C in sterile saline (0.9% NaCl) containing 0.1% Gentamycin. Cumulus oocyte complexes (COCs) were aspirated from 3–8 mm follicles using a syringe with a 18G needle. Only grade 1 and 2 COCs, as morphologically described by de Loos et al. (1989), were used [28]. Selected COCs were washed in phosphate-buffered saline (PBS) and in maturation medium (TCM 199 supplemented with 10% fetal calf serum (FCS) and 10 ng/mL epidermal growth factor (EGF)). Depending on the experiment, the maturation medium was modified with the addition of 5 ng/mL HSP70. The HSP70 dose was selected on the basis of HSP70 concentration that we measured in the peripheral blood of 164 heat stressed dairy cows [29]. COCs (*n* = 1933) were randomly allocated to 1 of 4 maturation protocols: 39 °C for 24 h without (group C39, *n* = 471) or with HSP70 (group H39, *n* = 353), for 6 h at 41 °C (from the 2nd to 8th hour of IVM) followed by 16 h at 39 °C in the presence (group H41, *n* = 704) or in the absence of HSP70 (group C41, *n* = 405). Maturation was carried out in an atmosphere of 5% CO₂, 20% O₂, with maximum humidity. 

After 24 h in IVM, matured COCs were inseminated with frozen–thawed, swim-up separated bull sperm at a final concentration of 1 × 10^6^ spermatozoa/mL. Gametes were co-incubated for 24 h in standard IVF medium at 39 °C, under an atmosphere of 5% CO₂, 20% O₂ with maximum humidity. Semen from the same Holstein bull and ejaculation was used for all experiments.

Approximately 20 h post insemination (pi), presumptive zygotes were denuded by gentle vortexing and cultured in groups of 25 in microdroplets (25 μL) under mineral oil. Zygotes were cultured for 9 days in synthetic oviductal fluid (SOF) supplemented with 5% FCS at 39 °C, in an atmosphere of 5% CO₂, 5% O₂ and 90% N₂, in maximum humidity.

Cleavage and blastocyst formation rates were recorded after stereo-microscopic observation at 48 h pi and on days 7, 8 and 9 pi, respectively. The in vitro embryo production experiment was carried out in 10 replicates. From 5 replicates, pools of 12 matured oocytes, with the respective cumulus cells and 12 D7 blastocysts, were snap-frozen in PBS in liquid nitrogen and stored at −80 °C, until gene expression analysis. The oocytes used for gene expression studies were mechanically denuded by sequential passages of the COCs through a fine glass pipette and were washed in PBS.

### 2.2. RNA Extraction and Reverse Transcription

Total RNA was extracted using PicoPure RNA Isolation Kit (Thermo Scientific, Waltham, MA, USA) according to the manufacturer’s protocol and was further treated with DNAfree DNA Removal kit (Thermo Scientific, Waltham, MA, USA) to remove any DNA residuals. RNA’s quantity and quality were assessed using a Qubit™ RNA BR Assay Kit. The cDNA synthesis was performed using Maxima H Minus First Strand cDNA Synthesis Kit (Thermo Scientific, Waltham, MA, USA), 15 ng of total RNA and a combination of oligodTs and random primers. cDNA samples were further diluted (1:2 for the oocytes, 1:5 for the cumulus cells and the blastocysts) and were stored at −80 °C.

### 2.3. Gene Expression Analysis

In oocytes, the gene expression of HSPA1A, HSP90AA1, HSPB11 (heat shock proteins), SOD2, GPX1 (antioxidants), G6PD (metabolism), BCL2 (cell cycle) and TLR2 (inflammation); in cumulus cells the expression of HSPA1A, HSP90AA1 (heat shock proteins), BCL2 (cell cycle), CPT1B, G6PD (metabolism), IGF1 (cell signaling), GSTP1 (antioxidant) and ATP1A1 (osmoregulation); and in blastocysts HSP90AA1, HSPA1A, HSF1 (regulation of HSPs’ expression) GPX1, GSTP1, PLAC8A (implantation), TLR2, ATP1A1, BAX, BCL2, DNMT3A (epigenetic regulation), AKR1B1 (metabolism) and IGF1 (signaling), were analyzed. Most of the primer pairs used in this study were from our previous study [25], and the additional pairs that were designed for this analysis are presented in Table 1. For the primer designment, PrimerBLAST and Primer3 were used [30], and their suitability was evaluated using Beacon Designer (http://www.premierbiosoft.com/qOligo/Oligo.jsp?PID=1 (accessed on 5 December 2019)).

qPCR was performed using KAPA SYBR FAST (Sigma Aldrich, Milwaukee, WI, USA) on an AB Step One Plus Mastercycler (Applied Biosystems, Waltham, MA, USA), in a 20 µL reaction containing 1.5 µL cDNA, gene-specific primers (300 nM final concentration) and 1× KAPA SYBR FAST qPCR Master Mix. The cycling conditions were: 5 min at 95 °C, followed by 40 cycles of 20 s at 95 °C and 20 s at 60 °C for annealing and extension. A melting curve step was performed for each reaction to ensure the specificity of the products. Samples were measured in duplicates and a threshold of ±0.2 in Cq differences between replicates was used to discard samples with discrepancies. Cq values were retrieved for each reaction by setting a constant threshold and the average efficiencies per gene were computed using the LinReg software, as proposed by Ramakers and his colleagues (2003) [31].

The relative gene expression was normalized using the geometric mean of three reference genes: YWHAZ, UBA52 and EEF1A1, which were evaluated using GeNorm, with the respective M value as an indicator of the gene expression stability across samples [32].

### 2.4. Statistical Analysis

Statistical analyses of in vitro embryo production (IVP) and gene expression studies were performed by R. In IVP, the results are expressed as means ± standard deviations. Data normality was checked using a Shapiro–Wilk test; there was homogeneity of variances, as assessed by Levene’s test of homogeneity of variance (*p* > 0.05). Two-way ANOVA was used to show differences between Temperature and HSP70. Moreover, we split the data into four strata to detect the actual relationship between variables. The objective of stratification was to fix the level of the potential effect modifier and produce groups within which the effect modifier did not vary. After that, we conducted an independent Student’s *t*-test to assess between-group differences with Temperature and HSP70 treatment in each phase. Significance was determined by a *p* value of <0.05.

The statistical analysis of differentially expressed genes (DEGs) was performed as follows:

1. A Two-way ANOVA test among all the four groups was used to detect the possible effect of the two factors (Temperature, HSP addition) along with their interaction in the differential gene expression. Pairwise comparisons were conducted between pairs of groups (C39, H39, C41, H41). We focused on the differences between the H and C groups, since our major question is to address the effect of HSP addition to the medium. The significant differences (*p*-values < 0.05) are presented in Supplementary Tables S2–S4.
2. Correlation coefficients were computed for each pair of genes in two groups (samples supplied with HSP70 and not supplied with HSP70) using the rcorr function, since correlated gene expression may be indicative of a similar regulation mechanism underlying gene expression. Coefficients were plotted using the corrplot function, where positive correlations are displayed in blue and negative correlations in red color. Color intensity and the size of the circle are proportional to the correlation coefficients.

## 3. Results

### 3.1. In Vitro Embryo Production

Cleavage rate in group C39 was significantly higher (*p* < 0.01) compared with groups H41 and C41 and tended to be higher compared with group H39 (*p* = 0.068). No other differences were detected among groups.

On days 7, 8 and 9 blastocyst formation rates in group C41 were significantly lower (*p* < 0.03) than in group C39, and on day 7, it tended (*p* = 0.06) to be lower than in group H39. Similarly, on day 9, the blastocyst formation rate in group C41 was significantly lower (*p* = 0.03) than in group H39. No difference was detected in the embryo yield between groups C39 and H41.

Details on in vitro embryo production are given in Table 2, and in Supplementary Table S1.

### 3.2. Gene Expression

The gene expression analyses were carried out on materials collected from groups C39, H39, C41 and H41.

#### 3.2.1. Oocytes

**Figure 1:**
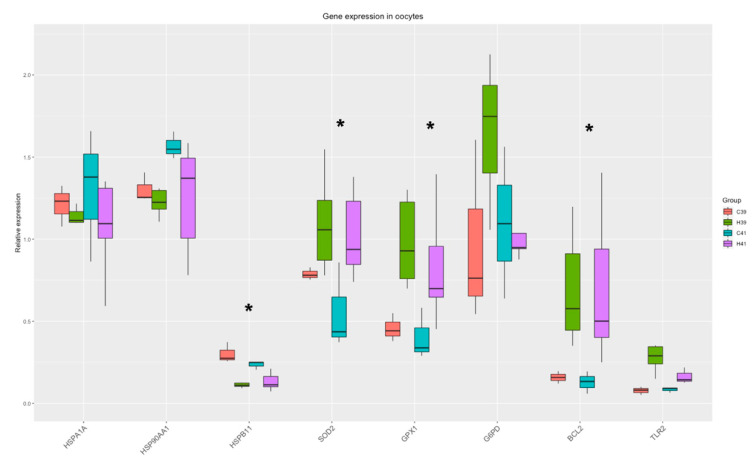
Gene expression in oocytes. Significant changes from the Two-Way ANOVA test are marked with asterisk (*).

The Two-Way ANOVA analysis revealed that the expression of four genes (*HSPB11*, *BCL2, GPX1, SOD2)* out of the eight measured were significantly (*p* < 0.035) altered by the presence of HSP70 in the medium and there was a strong tendency towards the differential expression of *G6PD* (*p*-value = 0.07). The relative expression of genes is presented in Figure 1.

**Figure 2:**
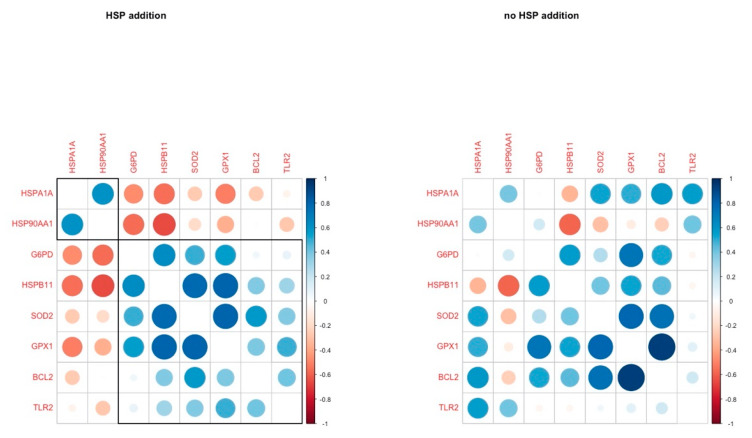
Pairwise correlation coefficients of genes under study in oocytes in H and C groups respectively.

The correlation analysis revealed three genes with a strong positive correlation (*HSPB11, SOD2, GPX1*) in the HSP addition group, and these genes were negatively correlated with two HSP genes (*HSP90AA1, HSPA1A*) in the same group. In the absence of HSP70, these correlations were absent, and *HSP90AA1* was negatively correlated with *HSPB11*, while *BCL2* was positively correlated with *GPX1* (Figure 2).

#### 3.2.2. Cumulus Cells

**Figure 3:**
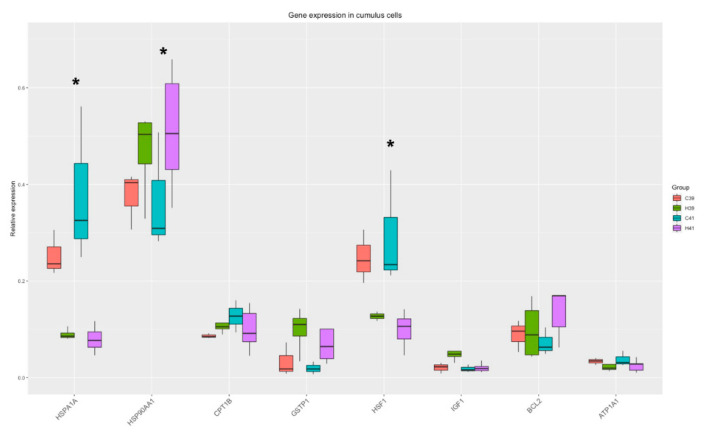
Gene expression in cumulus cells. Significant changes from the Two-Way ANOVA test are marked with *.

The Two-Way ANOVA analysis revealed that the expression of three genes (*HSPA1A, HSP90AA1, HSF1)* out of the eight measured were significantly altered by the presence of HSP70 in the medium and there was a strong tendency towards differential expression of *GSTP1* (*p*-value = 0.06). The relative expression of genes is presented in Figure 3. The significant differences between the C and the H groups are presented in Supplementary Table S3.

**Figure 4:**
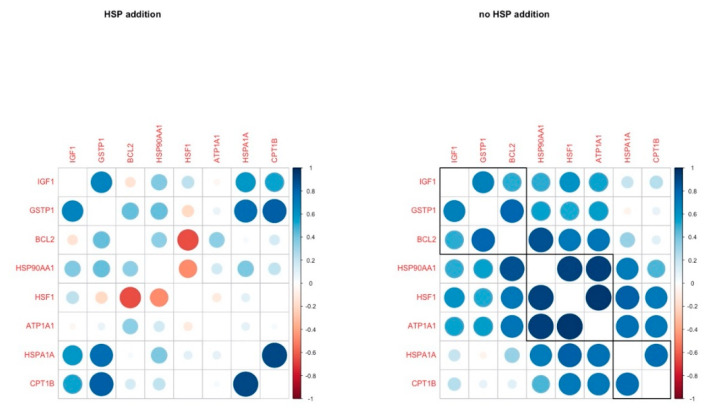
Pairwise correlation coefficients of genes under study in cumulus cells in H and C groups, respectively.

The correlation analysis revealed four genes with a strong positive correlation (r > 0.5, *IGF1, GSTP1, HSPA1A, CPT1B*) in the HSP addition group, while HSF1 was negatively correlated with *BCL2*. In the group without HSP addition, *HSP90AA1*, *HFS1*, *ATP1A1* were strongly and positively correlated, and these correlations were absent in the HSP70-treated group (Figure 4).

#### 3.2.3. Blastocysts

**Figure 5:**
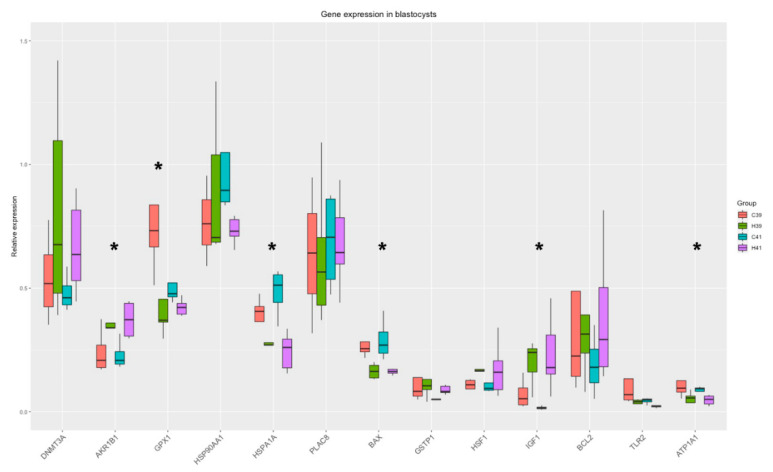
Gene expression in blastocysts. Significant changes from the Two-Way ANOVA test are marked with *.

HSP70 supplementation in IVM medium induced differential expression of 5 genes (*AKR1B1*, *GPX1*, *HSPA1A*, *IGF1*, *BAX, ATP1A1*), and there was a strong tendency towards differential expression in *GSTP1* (Figure 5). The significant differences between the C and the H groups are presented in Supplementary Table S4.

**Figure 6:**
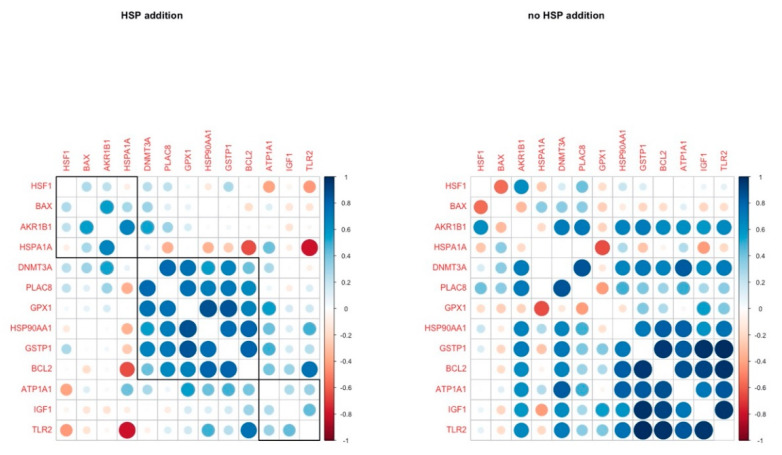
Pairwise correlation coefficients of genes under study in blastocysts in H and C groups respectively.

HSP addition led to a positive correlation in the gene expression of 6 genes (*DNMT3A, PLAC8, GPX1, HSP90AA1, GSTP1, BCL2),* while most of these correlations were absent in the other group. However, another group of genes (*HSP90AA1, GSTP1, BCL2, ATP1A1, IGF1, TLR2)* were tightly correlated in the group without the addition of HSP70. *HSP90AA1* and *GSTP1* were constantly positively correlated in both groups (Figure 6).

## 4. Discussion

This study shows for the first time that the presence of exogenous HSP70 in the IVM medium can blunt the deleterious effects of temperature rise on blastocyst yield and preserve oocyte and embryo quality by altering the expression pattern of a number of important genes. In addition, we provide evidence that in comparison to the cumulus cells, the oocyte is more responsive to HSP70 addition, and the compensation effects to the detrimental heat increase remain up to the blastocyst stage.

Here, we confirmed our previous observation [25] that a short lasting exposure of maturing oocytes to heat stress causes a significant reduction in cleavage rate. The addition of HSP70 in the IVM medium could not alleviate the effect of high temperature to cleavage rate. Our hypothesis that an addition of HSP70 in the maturation medium will improve the cleavage rate was not confirmed; on the contrary, HSP70 supplementation without a temperature rise (H39) led to a slightly decreased (*p* = 0.068) cleavage rate in comparison to C39. A good body of evidence suggests that temperature elevation, even for a short period during IVM, brings about an impaired fertilizing capacity and a reduced embryo production rate [33,34,35]. HSP70 acting on microtubules contributes to the stabilization of meiotic spindle formation in the oocyte. While most HSPs are synthesized under normal conditions, HSP70 is synthesized exclusively under stress [9]. Hence, it could be postulated that under physiological conditions, the presence of HSP70 in the IVM medium was construed as a potential insult stimulus by the oocyte and/or the cumulus cells, triggering defense mechanisms that partly suppressed the cleavage rate.

As was expected, temperature rise during IVM substantially reduced blastocyst formation rate in group C41. This result is consistent with established and published findings [25,36,37,38] and the underlying mechanisms are extensively discussed in our previous paper [25]. Despite being under heat stress conditions, the blastocyst yield was restored in the presence of HSP70, and it did not differ at any time point from that of group C39. Obviously, this was attributed to the protective role of HSP70. In general, the presence of HSP70 in the maturation medium altered the expression of HSPs (*HSP90AA1, HSPB11*) in oocytes, regardless of the temperature. *HSP90AA1* encode for a molecular chaperon that is involved in the proper folding, transport and stability of specific target proteins by use of an ATPase activity, modulated by co-chaperones [39]. *HSP90AA1* is also involved in many other cellular functions, such as cell signaling, transcription, kinase regulation, and DNA replication and repair [40]. HSP72, encoded by *HSPA1A*, also prevents cell death in multiple ways [41]. On the other hand, *HSPB11* that encodes for a small heat shock protein acts as a molecular chaperone that prevents apoptotic cell death [42] via an HSP90-mediated mechanism that stabilizes the mitochondrial membrane [43]. In our previous study [25], the elevation of temperature from 39 to 41 °C for six hours led to an increased expression of *HSPB11* in heat-stressed oocytes, while there was no significant difference in *HSPA1A* and *HSP90AA1*. This finding is in accordance with another study where HSPB11 was the only one among the 16 HSP genes studied that was significantly altered in heat-stressed bovine embryos [44]. Thus, the HSP70 supplementation alone appears to trigger a stronger response to the treated oocytes, which is further supported by the elevated expression of *SOD2* and *GPX1* (antioxidants). *SOD2* is a member of the iron/manganese superoxide dismutase family that catalyzes the conversion of the superoxide radicals of oxidative phosphorylation to hydrogen peroxides and diatomic oxygen [45]. *GPX1* encodes a protein that belongs to the glutathione peroxidase family, members of which transform hydrogen peroxides by glutathione to water [46]. *GPX1* expression increased in both the oocytes and the blastocysts in the presence of HSP70 addition (H39), compared with the oocytes and blastocysts matured at 39 °C (C39), indicating that HSP70 is higher in the hierarchy of the heat response cascade than the protective mechanism against the temperature rise induced oxidative stress. HSP70 supplementation also led to a significant increase in the expression of *BCL2* regardless of the temperature. *BCL2* acts as a protective molecule during heat stress through its anti-apoptotic role, and it has been also been found to be over-expressed in studies of heat-stressed cows [47,48,49,50]. *BCL2* encodes an anti-apoptotic protein, which functions as a regulator of cell death by inhibition of the action of pro-apoptotic proteins. Its expression has been associated with high embryo quality [51,52].

Furthermore, the HSP70 supplementation led to a tight coordination of the expression of *G6PD, HSPB11, SOD2* and *GPX1.* It appears that HSP70 is closely linked to the regulation of the oxidative stress mechanism along with HSPB11, and it brings the three antioxidant genes (*G6PD, SOD2, GXP1*) under the same regulation cluster.

In cumulus cells, HSP70 supplementation resulted in modifying the expression of three genes, namely *HSPA1A, HSP90AA1* and *HSF1*. Heat shock factors (HSFs), which are encoded by the *HSF1* gene, are responsible for the induction of HSPs synthesis in stressful conditions [11]. In heat stress conditions, HSFs are activated, enter the nucleus and attach to the heat shock elements (HSEs). This interaction leads to the transcription of HSPs. Exogenous administration of HSP70 possibly leads the cell to a reflective need for modification in the production of HSPs. *HSPA1A* belongs to the HSP70 family of proteins, so we assume that there is a negative feedback in the expression of these proteins due to the external administration of HSP70 in the medium, while HSP90AA1 is a member of the HSP90 family, and it is up regulated to fulfill its cytoprotective roles as mentioned above.

The coordinated patterns of gene expression in cumulus cells are indicative of the processes taking place in every group. Comparing the gene coordination patterns across groups, it was revealed that the expression of IGF1 is tightly coordinated with the expression of *CPT1B* and *GSTP1* regardless of the temperature when HSP70 is supplemented. *CPT1B* encodes for carnitine palmytoltransferase 1, a rate-limiting enzyme of fatty acid β-oxidation, which is important for the progression of meiosis and the developmental competence of the oocytes [53]. Its close coordination with IGF1 indicates that it might fall within the spectrum of IGF1 regulation of growth mechanisms. It is noteworthy that glutathione S-transferase (GSTP1), key in redox scavenging in the cells, is so tightly linked to energy provision and growth genes. GSTP1 is known to exhibit an anti-apoptotic function through different pathways [54], yet to our knowledge it has not linked before to growth mechanisms. Moreover, *HSF1* and BCL2 are showing a strong negative correlation, since *HSF1* expression is downregulated due to the HSP70 supplementation, while *BCL2* expression is upregulated (though not significantly), especially in the H41 group as reflected in pairwise comparisons (Supplementary Table S3), acting as an anti-apoptotic molecule as mentioned above.

HSP70 supplementation altered the expression of *AKR1B1, IGF1*, *GPX1, HSPA1A, BAX* and *ATP1A1* (Figure 5), while GSTP1 also showed significant differences between the C and the H groups. The expression of *AKR1B1*, which protects against toxic aldehydes derived from lipid peroxidation, is upregulated in HSP70 presence, indicating the protective effects of HSP70. Furthermore, *IGF1* is responsible for the regulation of growth mechanisms and its upregulation can highlight the growth potential of the blastocysts. On the other hand, *BAX* is encoding for an apoptosis activator and is downregulated in the H groups, thus protecting the cells from apoptosis. *HSPA1A* is constantly downregulated in H groups along all cell types (oocytes, cumulus cells, blastocysts) and a possible explanation has already been proposed.

In the HSP70 supplementation group, *BCL2* and *GPX1* cluster with *GSTP1*, *DNMT3A*, *HSP90AA1* and *PLAC8A*. These genes are connected to blastocyst quality and survival (*BCL2* is anti-apoptotic, *GPX1* and *GSTP1* are antioxidants, *PLAC8A* is responsible for normal embryo implantation and *DNMT3A* is responsible for epigenetic reprogramming of the embryo after embryo genome activation (EGA)) and their coordinated expression may orchestrate the blastocysts’ survival in the presence of the anti-inflammatory signals (extracellular HSP70). This cluster of correlation did not survive on the C group; *BCL2* and *GSTP1* expression was strongly correlated with *ATP1A1*, *IGF1*, and *TLR2*. These genes are participating in the response to stress (elevated temperature and anti-inflammatory signals), while they are also regulators of apoptosis and growth. As HSP70 contributed to increased embryo yield in H41, it could be hypothesized that it was due to the enhancement of all these protective mechanisms that the fertilization and development of oocytes which lacked some type of inherent thermotolerance to the blastocyst stage was inevitably permitted.

In conclusion, it was demonstrated that the external supplementation of HSP70 can offset the deleterious effects of heat stress on embryo production. In this study, the presence of exogenous HSP70 acted protectively for oocytes and cumulus cells and formed blastocysts, by intercepting apoptosis, promoting signal transduction and increasing the antioxidant protection of the embryo.

## Figures and Tables

**Table 1:** Primer information: sequence, size of the amplified fragments of transcripts and accession number.

Gene Name	Gene Description	Forward Primer	Reverse Primer	Product Size (bp)
*TLR2*	Toll like receptor 2	GCTGCCATTCTGATTCTGCT	GCCACTCCAGGTAGGTCTTG	103
*BCL2*	BCL2 apoptosis regulator	CCCTGTTTGATTTCTCCTGGC	CTGTGGGCTTCACTTATGGC	107
*HSF1*	Heat shock transcription factor 1	ATGAAGCACGAGAACGAGGC	GCACCAGCGAGATGAGGAACT	112
*ATP1A1*	ATPase Na+/K+ transporting subunit alpha 1	CGCCAGGGTTTATCCAGTT	AGGGGAAGCCAGTTTTTGTT	80
*IGF1*	Insulin like growth factor 1	TCACATCCTCCTCGCATCTCTT	AGCATCCACCAACTCAGCC	107
*BAX*	BCL2 associated X, apoptosis regulator	TTTGCTTCAGGGTTTCATCC	CGCTTCAGACACTCGCTCAG	120

**Table 2:** Cleavage and blastocyst formation rates (mean ± SD) in four groups of COCs matured in vitro at 39 °C without (group C39) or with HSP70 (group H39), at 41 °C for 6 h from the 2nd to 8th hour of IVM without (group C41) or with HSP70 (group H41).

			Blastocysts		
Group	COCs	Cleaved (%)	Day 7 (%)	Day 8 (%)	Day 9 (%)
C39	519	438 ^a^ (84.4 ± 4.5)	154 ^a^ (29.7 ± 6.6)	172 ^a^ (33.1 ± 6.0)	179 ^a^ (34.5 ± 7.6)
H39	353	286 ^a,b^ (81.0 ± 7.5)	94 ^ab^ (26.6 ± 4.4)	111 ^ab^ (31.4 ± 6.0)	122 ^ab^ (34.5 ± 6.5)
C41	508	401 ^b,c^ (78.8 ± 6.8)	102 ^b^ (20.1 ± 3.7)	123 ^b^ (24.2 ± 7.9)	129 ^c^ (25.5 ± 9.0)
H41	704	551 ^b,c^ (78.2 ± 7.4)	186 ^ab^ (26.4 ± 10.4)	226 ^ab^ (32.1 ± 11.2)	235 ^abc^ (33.4 ± 11.4)

Within columns, values marked with different superscripts (^a,b,c^) differ significantly (*p* < 0.05).

## Data Availability

The data presented in this study are available upon reasonable request from the corresponding author.

## Supplementary Materials

The following are available online at https://www.mdpi.com/article/10.3390/ani11061794/s1, Figure S1: Distribution of blastocysts according to their developmental stage in four groups of COCs matured in vitro at 39 °C without (group C39) or with HSP70 (group H39), at 41 °C for 6 hours from the 2nd to 8th hour of IVM without (group C41) or with HSP70 (group H41), Table S1: Distribution of blastocysts according to their developmental stage in four groups of COCs matured in vitro at 39^ο^ C without (group C39) or with HSP70 (group H39), at 41 °C for 6 hours from the 2nd to 8th hour of IVM without (group C41) or with HSP70 (group H41), Table S2: Significant differences in gene expression between C and H groups in oocytes, Table S3: Significant differences in gene expression between C and H groups in cumulus cells, Table S4: Significant differences in gene expression between C and H groups in blastocysts.
