# Supplementary material for: Heat Shock Protein 70 Improves In Vitro Embryo Yield and Quality from Heat Stressed Bovine Oocytes
Source: Animals (Basel). 2021 Jun 16;11(6):1794. doi: 10.3390/ani11061794 (PMC8235242; doi:10.3390/ani11061794)
Supplement: Supplementary file 1 [file animals-11-01794-s001.zip › animals-1209867-supplementary.pdf]

Table S1. Distribution of blastocysts according to their developmental stage in four groups of COCs matured *in vitro* at 39° C without (group C39) or with HSP70 (group H39), at 41°C for 6 hours from the 2nd to 8th hour of IVM without (group C41) or with HSP70 (group H41).

	**Day 7 blastocysts**				**Day 8 blastocysts**				**Day 9 blastocysts**			
Embryostage	EaB	B	ExB	HB	EaB	B	ExB	HB	EaB	B	ExB	HB
**C39**	48	106	0	0	7	147	12	6	0	70	87	22
**H39**	32	62	0	0	17	82	10	2	1	45	61	15
**C41**	39	63	0	0	10	108	5	0	3	87	27	12
**H41**	60	126	0	0	12	189	17	8	3	111	96	25

EaB: Early blastocyst; B: blastocyst; ExB Expanded blastocyst; HB: hatched blastocyst

Table S2. Significant differences in gene expression between C and H groups in oocytes.

Gene name	C group	H group
HSP90AA1	C41	H41
HSP90AA1	C41	H39
HSPB11	C39	H39
HSPB11	C39	H41
HSPB11	C41	H41
HSPB11	C41	H39
SOD2	C39	H39
SOD2	C39	H41
GPX1	C39	H39
GPX1	C39	H41
GPX1	C41	H41
GPX1	C41	H39
G6PD	C39	H39
G6PD	C41	H39
BCL2	C39	H39
BCL2	C39	H41
BCL2	C41	H41
BCL2	C41	H39

Table S3. Significant differences in gene expression between C and H groups in cumulus cells.

Gene name	C group	H group
HSPA1A	C39	H39
HSPA1A	C39	H41
HSPA1A	C41	H41
HSP90AA1	C39	H41
GSTP1	C41	H39
GSTP1	C41	H41
HSF1	C39	H41
IGF1	C39	H39
IGF1	C41	H39
BCL2	C39	H41
BCL2	C41	H41

Table S4: Significant differences in gene expression between C and H groups in blastocysts

Gene name	C group	H group
HSPA1A	C39	H41
	C41	H41
AKR1B1	C39	H39
AKR1B1	C39	H41
AKR1B1	C41	H39
AKR1B1	C41	H41
GSTP1	C39	H39
GSTP1	C41	H41
GSTP1	C41	H39
IGF1	C39	H39
IGF1	C39	H41
IGF1	C41	H39
IGF1	C41	H41
ATP1A1	C39	H41
ATP1A1	C41	H41
